# Humanistic and economic burden associated with depression in the United States: a cross-sectional survey analysis

**DOI:** 10.1186/s12888-022-04165-x

**Published:** 2022-08-11

**Authors:** Saundra Jain, Shaloo Gupta, Vicky W. Li, Ellison Suthoff, Alix Arnaud

**Affiliations:** 1 University of Texas, Austin, USA; 2 Cerner Enviza, 51 Valley Stream Pkwy, Malvern, PA 19355 USA; 3 Sage Therapeutics, Inc., Cambridge, MA USA

**Keywords:** Anxiety, Depression, Healthcare resource utilization, Quality of life, Sleep disorder, Work productivity

## Abstract

**Background:**

Depression (major depressive disorder [MDD]) affects the functioning of patients in many facets of life. Very few large-scale studies to date have compared health and economic related outcomes of those with versus without depression, and across various depression severity groups. We aimed to evaluate humanistic and economic burden in respondents with and without depression diagnosis, and across symptom severity groups.

**Methods:**

Data from the 2017 US National Health and Wellness Survey (NHWS) were utilized. Of the adult respondents (*N* = 75,004), 59,786 were < 65 years old. Respondents not meeting eligibility criteria were excluded (e.g., those self-reporting bipolar disorder or experiencing depression in past 12 months but no depression diagnosis). Overall, data from 39,331 eligible respondents (aged 18–64 years) were analyzed; and comprised respondents ‘with depression diagnosis’ (*n* = 8853; self-reporting physician diagnosis of depression and experiencing depression in past 12 months) and respondents ‘without depression diagnosis’ (*n* = 30,478; no self-reported physician diagnosis of depression and not experiencing depression). Respondents with depression were further examined across depression severity based on Patient Health Questionnaire-9 (PHQ-9). Outcome measures included health-related quality-of-life (HRQoL; Medical Outcomes Study 36-item Short Form [SF-36v2]: mental and physical component summary [MCS and PCS]; Short-Form 6 Dimensions [SF-6D]; and EuroQol 5 Dimensions [EQ-5D]), work productivity and activity impairment (WPAI), and health resource utilization (HRU). Multivariate analysis was performed to examine group differences after adjusting covariates.

**Results:**

Respondents with depression diagnosis reported significantly higher rates of diagnosed anxiety and sleep problems versus those without depression (for both; *P* < 0.001). Adjusted MCS, PCS, SF-6D, and EQ-5D scores were significantly lower in respondents with depression versus those without depression (all *P* < 0.001). Consistently, respondents with depression reported higher absenteeism, presenteeism, and overall WPAI, as well as greater number of provider visits, emergency room visits, and hospitalizations compared with those without depression (all *P* < 0.001). Further, burden of each outcome increased with an increase in disease severity.

**Conclusions:**

Diagnosed depression was associated with lower health-related quality-of-life and work productivity, and higher healthcare utilization than those without depression, and burden increased with an increase in symptom severity. The results show the burden of depression remains high even among those experiencing minimal symptoms.

**Supplementary Information:**

The online version contains supplementary material available at 10.1186/s12888-022-04165-x.

## Background

Depression is a common mental disorder in the United States (US) [1]. Major depressive disorder (MDD) is a form of depression, defined as the presence of ≥ 5 of the following symptoms in the same 2-week period (with ≥ 1 symptom being depressed mood or anhedonia): (1) depressed mood, (2) marked loss of interest or pleasure in everyday activities (anhedonia), (3) significant change in weight or change in appetite, (4) insomnia or hypersomnia, (5) psychomotor agitation or retardation, (6) fatigue or loss of energy, (7) feelings of worthlessness or guilt, (8) diminished ability to think or concentrate, and (9) recurrent thoughts of death or suicidal ideation or suicide attempt [2]. Additional criteria that must be met for an MDD diagnosis are: the symptoms cause significant distress/impairment, the episode is not attributable to a substance’s physiological effects, occurrence of episode is not explained by other psychotic disorders, and absence of a manic episode/hypomanic-like episode [2]. According to the Global Burden of Disease study (1990 to 2017), the incident cases of MDD increased by 49.29% from 162 to 241 million globally [3]. As per the National Survey on Drug Use and Health report, 21.0 million adults had experienced ≥ 1 major depressive episode in 2020, representing 8.4% of the adult population in the US; prevalence was higher among female adults than males and in individuals aged 18-49 years than those aged ≥ 50 years [4].

Numerous studies have reported on the detrimental impact of MDD on overall health-related quality of life (HRQoL) in terms of interpersonal relationships, and psychological and physical functioning, noting that the psychological impairment can persist even after remission of MDD symptoms [5–9]. According to an analysis of quality of life (QoL) data from the Sequenced Treatment Alternatives to Relieve Depression (STAR*D) trial, < 3% of untreated patients with MDD reported “within-normal” QoL and approximately 50% of treated patients continued to experience “severely-impaired” QoL [9]. Moreover, another analysis of the STAR*D trial data reported a decrease in both response and remission rates with each step of treatment [10].

Further, previous studies have shown that depression affects work productivity [11] and increases healthcare resource utilization (HRU), leading to higher direct and indirect costs with increasing severity of depression [12, 13]. The economic burden in adults with MDD is high, with 37.9% increase ($237 billion to $326 billion) in all costs from 2010 to 2018 in the US [14]. This burden includes the costs attributed to not only MDD management (37.0%) but also comorbid conditions (63.0%) [14].

A nationally representative survey (2012–2013) conducted in the US reported that among respondents with lifetime MDD (*n* = 7432), the lifetime prevalence of comorbid anxiety disorder was 37.3% and substance use disorder was 57.9% [15]. Patients with both MDD and anxiety disorders incur higher healthcare costs as compared to those with either disorder alone [16]. Considering the humanistic and economic impact MDD has on an individual, it is important to not only treat depressive symptoms but also focus on improving overall QoL in the presence of comorbidities.

Although previous studies have evaluated the burden of MDD on QoL [5, 7–9], work productivity and activity impairment (WPAI) [11–13], HRU [12], and health economic outcomes across depression severity groups [5, 7, 12, 13], few studies to date have compared the outcomes of those without depression to those with depression or across various depression severity groups in a large sample size in the US [12, 14]. Thus, there is a need for more recent data in the US population. The objective of this large, cross-sectional study was to evaluate the humanistic and economic burden of depression in the US by comparing outcomes (HRQoL, WPAI, and HRU) of respondents with depression diagnosis versus without depression diagnosis and also across symptom severity groups.

## Methods

### Study design and data source

This study was conducted using data from the 2017 US National Health and Wellness Survey (NHWS; *N* = 75,004). The NHWS is a self-administered, internet-based survey of a sample of adults (aged ≥ 18 years) that provides “real world” patient-level information over 165 therapeutic conditions. Potential respondents for the survey are recruited through a general-purpose web-based consumer panel. The panel recruits its members via opt-in e-mails, co-registration with panel partners, e-newsletter campaigns, banner placements, and affiliate networks. All the respondents who explicitly agreed to be a panel member registered through a unique e-mail address and completed an in-depth demographic registration profile. A quota sampling procedure (using data from the Current Population Survey of the US Census) was used to ensure that the final NHWS sample was representative of the US’ adult population in 2017 with respect to age, gender, and race/ethnicity. Informed consent was obtained from all the respondents and all parties ensured protection of patients’ personal data. The study protocol and questionnaire were reviewed by the Pearl Institutional Review Board and granted exemption status.

### Study sample

**Fig. 1:**
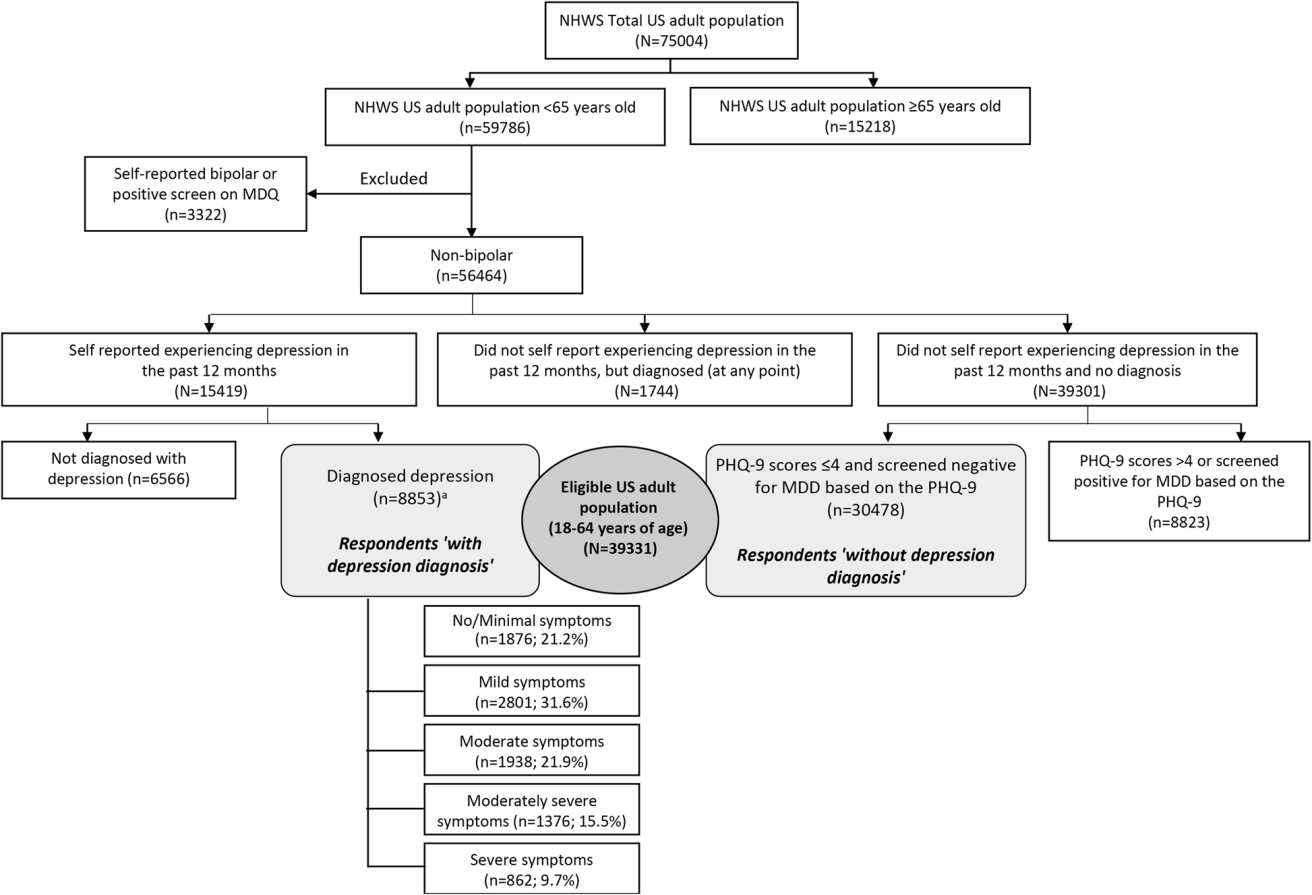
Eligible US NHWS sample for participants 18–64 years of age ^a^Patients with depression diagnosis were stratified by PHQ-9 score at time of survey. MDQ, mood disorder questionnaire; NHWS, National Health and Wellness Survey; PHQ-9, the Patient Health Questionnaire 9; US, United States

Respondents aged 18-64 years ‘with depression diagnosis’ (*n* = 8853) or ‘without depression diagnosis’ (*n* = 30,478) were included in the analysis. Respondents with depression diagnosis: those who self-reported physician diagnosis of depression and reported experiencing depression in the past 12 months) [2]. These respondents were further stratified by severity of depression as determined by Patient Health Questionnaire-9 (PHQ-9) scores: none/minimal (score = 0–4; *n* = 1876), mild (score = 5–9; *n* = 2801), moderate (score = 10–14; *n* = 1938), moderately severe (score = 15–19; *n* = 1376), or severe (score = 20–27; *n* = 862). Respondents without depression diagnosis: those who had no self-reported physician diagnosis of depression, reported not experiencing depression in the past 12 months, and had PHQ-9 scores ≤ 4 [17] (Fig. 1). Respondents diagnosed with bipolar disorder and those who reported not experiencing depression in the past 12 months but had a diagnosis were excluded from the study.

### Measures

#### Demographics and health characteristics

Demographic variables including age, gender, employment status, race/ethnicity, marital status, education, household income, insurance status, body mass index (BMI), smoking status, alcohol use, exercise behavior, and Charlson Comorbidity Index (CCI) were collected. The CCI represents a weighted sum of multiple comorbid conditions predictive of mortality with greater scores indicating greater comorbid burden on the patient [18]. Disease-specific diagnoses including depression, anxiety, and sleep difficulties were also analyzed.

#### Depression symptoms, anxiety and sleep problems

Depression symptoms assessed that prompted respondents to see their doctor included self-reported depressed mood and other emotional problems, changes in eating and sleep patterns, mental changes (e.g., forgetfulness, difficulty thinking, difficulty concentrating), and social and physical problems. Sleep problems including self-reported difficulty falling asleep, difficulty staying awake, daytime sleepiness, leg cramps/leg problems, night sweats/hot flashes, and poor quality of sleep were evaluated. Anxiety was assessed according to the self-reported diagnoses of anxiety disorders and self-reported experiences of anxiety. Additionally, anxiety was measured by the Generalized Anxiety Disorder-7 (GAD-7) scale (Supplementary Table 1) [19].

#### Health-related quality of life (HRQoL) and health utilities

##### Short Form Survey Instrument version 2 (SF-36v2)

HRQoL was assessed using the SF-36v2 [20], which is a multipurpose, generic health status instrument comprised of 36 questions. The instrument is designed to report eight health domains (Physical Functioning, Role-Physical, Bodily Pain, General Health, Vitality, Social Functioning, Role-Emotional, and Mental Health) and two summary scores (Physical Component Summary [PCS] and Mental Component Summary [MCS]). Each domain and PCS and MCS scores are normed to a mean of 50 and a standard deviation of 10 for the US’ population. Higher scores are indicative of better health status [20]. SF-36v2 related parameters were studied based on past 4 weeks health status. Additionally, health state utility index was calculated using the Short-Form 6 Dimensions (SF-6D) form. The SF-6D is a preference-based single index measure for health using general population values and provides scores on a theoretical 0–1 scale with higher scores indicating better health status [21].

##### EuroQol 5-Dimension Health Questionnaire

The EuroQol 5-Dimension Health Questionnaire (EQ-5D-5L) [21] consists of a descriptive system (EQ-5D) and a visual analogue scale (EQ VAS). The descriptive system is composed of five dimensions: mobility, self-care, usual activities, pain/discomfort, and anxiety/depression. The EQ VAS (score: 0 to 100) indicates the respondent’s self-rated health, with the endpoints being 'Best imaginable health state' (score = 100) and 'Worst imaginable health state' (score = 0). Lower overall scores on the EQ-5D-5L health utilities are indicative of higher disability. The most recent version with 5-point rating scales for each dimension was used in this study [22]. The EQ-5D-5L utility scores were calculated by mapping the five-level descriptive system (EQ-5D-5L) onto the three-level value set (EQ-5D-3L) using the mapping (“crosswalk”) approach developed by van Hout et al. [23]. Health states were mapped using country-specific value set.

#### Work productivity and activity impairment (WPAI)

Work productivity loss was measured using the WPAI questionnaire [24], a six-item validated instrument which consists of four metrics: absenteeism (the percentage of work time missed because of one's health in the past seven days), presenteeism-related impairment (the percentage of impairment experienced while at work in the past seven days because of one's health), overall work productivity loss (an overall impairment estimate that it is a combination of absenteeism and presenteeism), and activity impairment (the percentage of impairment in daily activities because of one's health in the past seven days). Only respondents who reported being employed full-time or part-time provided data for absenteeism, presenteeism, and overall work impairment; all respondents reported data for activity impairment.

#### Health-resource utilization (HRU)

Healthcare utilization was defined by the number of healthcare provider (HCP) visits (e.g., general practitioner, internist, cardiologist, gynecologist, etc.), the number of emergency room (ER) visits, and the number of times hospitalized in the past six months. All outcome measures and scales [19–21, 24, 25] used in this study are detailed in Supplementary Table 1.

### Statistical analyses

Chi-square and analysis of variance (ANOVA) tests were used to determine the significant differences for categorical variables and continuous variables, respectively. These results served to characterize differences between respondents with and without a depression diagnosis as well as between no/minimal, mild, moderate, moderately severe, and severe diagnosed depression and informed the selection of covariates for multivariable models.

Generalized linear models (GLMs) were used to control for demographic, health characteristic and comorbidity variables to compare HRQoL, WPAI, and HRU between respondents with and without a depression diagnosis and across symptom severity among respondents with a depression diagnosis. Only variables that were statistically significant in the bivariate analysis and had clinical importance were included in the regression models. GLMs with a negative binomial distribution were used for skewed data (e.g., WPAI and HRU).

The covariates included in the multivariable models were: Age (continuous), gender (male vs. female), ethnicity (black, hispanic, other vs. white [reference]), marital status (single, decline to answer vs. married/living with partner [reference]), education (less than college, decline to answer vs. college education [reference]), income (< 50 k, 50-75 k, decline to answer vs. 75 k + [reference]), employment (employed vs. not), insured (yes vs. no), BMI (underweight, overweight, obese [combined obese and morbidly obese], decline to answer vs. normal weight [reference]), smoking (former, current vs. never smoked), CCI, and individual comorbidities. The individual comorbidities included: Diagnosed with anxiety, nasal allergies/hay fever, allergies, pain, hypertension, high cholesterol, migraine, generalized anxiety disorder, heartburn, social anxiety disorder, gastroesophageal reflux disease, asthma, arthritis, panic disorder, acne, post-traumatic stress disorder, urinary tract infection, irritable bowel syndrome, dry eye, sleep apnea, eczema, thyroid problem, diabetes, yeast infection, and bladder control condition.

In bivariate analyses, comparisons were made for: (a) ‘with depression diagnosis’ versus ‘without depression diagnosis’ groups, and (b) across all severity groups using an overall omnibus test. In multivariable analyses, comparisons were made for: (a) ‘with depression diagnosis’ group versus ‘without depression diagnosis’ group (reference), and (b) mild, moderate, moderately severe, or severe groups versus ‘no/minimal’ symptoms severity group (reference). P-value less than 0.05 was considered statistically significant.

## Results

The NHWS total US adult population consisted of 75,004 respondents, of which 59,786 respondents were < 65 years old. Of these, respondents who self-reported bipolar disorder/screened positive on the mood disorder questionnaire were excluded (*n* = 3322). Of the non-bipolar respondents (*n* = 56,464): (a) 15,419 respondents self-reported experiencing depression in the past 12 months, of which 8853 respondents reported diagnosed depression; (b) 39,301 respondents did not self-report experiencing depression in the past 12 months and reported no depression diagnosis, of which 30,478 respondents had PHQ-9 scores ≤ 4 and screened negative for MDD based on the PHQ-9; and (c) 1744 respondents did not self-report experiencing depression in the past 12 months but were diagnosed (at any point) (Fig. 1).

This study included a total of 39,331 eligible respondents (aged 18–64 years), consisting of 8853 respondents who reported depression diagnosis and 30,478 respondents who reported no depression diagnosis. Respondents with depression diagnosis were further stratified by PHQ-9 scores as indicated in Fig. 1. Among these respondents, 47.2% had moderate to severe symptoms, 31.6% had mild symptoms, and 21.2% had no/minimal depression symptoms at the time of survey.

### Demographics and health characteristics

Data on the demographic and health characteristics, depression symptoms, anxiety and sleep problems, and prescription use for treating anxiety disorders or sleep problems collected from NHWS respondents are reported in Table 1. Respondents with depression diagnosis were more likely to be female, non-Hispanic white, single, have less than a college education, have lower annual household income, and less likely to be employed full-time than respondents without a depression diagnosis (all *P* < 0.001) (Table 1). Those with severe symptoms tended to be younger, female, single, have less than a college education, have lower annual household income, and less likely to be employed full-time compared to those with no/minimal symptoms (all omnibus *P* < 0.001). Furthermore, respondents with severe symptoms also tended to be obese, less likely to exercise regularly, and have higher CCI scores compared to those with no/minimal symptoms (all omnibus *P* < 0.001).

**Table 1:** Demographics, health characteristics of respondents with depression versus without depression diagnosis and across severity groups

**Variable**	**Respondents without depression diagnosis**^b*^ n = 30,478	**Respondents with depression diagnosis**^**a**^					
		Total (all symptom levels)^*^ n = 8853	**No/minimal symptoms**^**#**^ *n* = 1876	**Mild symptoms**^**#**^ *n* = 2801	**Moderate symptoms**^**#**^ *n* = 1938	**Moderately severe symptoms**^**#**^ *n* = 1376	**Severe symptoms**^**#**^ *n* = 862
**Demographic characteristics**							
Age (years), mean ± SD	43.9 ± 13.6	39.9 ± 14.1	42.1 ± 13.8	41.0 ± 14.0	39.0 ± 14.2	37.8 ± 13.8	37.1 ± 14.1
Female, n (%)	15600 (51.2)	6282 (71.0)	1265 (67.4)	1968 (70.3)	1416 (73.1)	993 (72.2)	640 (74.2)
Race/ethnicity, n (%)							
Non-Hispanic white	17860 (58.6)	5817 (65.7)	1244 (66.3)	1918 (68.5)	1259 (65.0)	865 (62.9)	531 (61.6)
Non-Hispanic black	3866 (12.7)	818 (9.2)	197 (10.5)	241 (8.6)	169 (8.7)	134 (9.7)	77 (8.9)
Hispanic	3373 (11.1)	1184 (13.4)	228 (12.2)	348 (12.4)	273 (14.1)	186 (13.5)	149 (17.3)
Other ethnicity	5379 (17.6)	1034 (11.7)	207 (11.0)	294 (10.5)	237 (12.2)	191 (13.9)	105 (12.2)
Marital status, n (%)							
Single	12301 (40.4)	4825 (54.5)	936 (49.9)	1501 (53.6)	1066 (55.0)	789 (57.3)	533 (61.8)
Married/living with partner	18096 (59.4)	4015 (45.4)	937 (49.9)	1299 (46.4)	867 (44.7)	587 (42.7)	325 (37.7)
Decline to answer	81 (0.3)	13 (0.1)	3 (0.2)	1 (0.0)	5 (0.3)	0 (0.0)	4 (0.5)
Education level, n (%)							
Less than college educated	13140 (43.1)	5437 (61.4)	1003 (53.5)	1649 (58.9)	1240 (64.0)	930 (67.6)	615 (71.3)
College educated	17289 (56.7)	3401 (38.4)	866 (46.2)	1149 (41.0)	694 (35.8)	446 (32.4)	246 (28.5)
Decline to answer	49 (0.2)	15 (0.2)	7 (0.4)	3 (0.1)	4 (0.2)	0 (0.0)	1 (0.1)
Annual household income, n (%)							
< $25 K	3420 (11.2)	2185 (24.7)	349 (18.6)	645 (23.0)	491 (25.3)	427 (31.0)	273 (31.7)
$25 K to < $50 K	5835 (19.1)	2499 (28.2)	464 (24.7)	798 (28.5)	563 (29.1)	418 (30.4)	256 (29.7)
$50 K to < $75 K	5775 (18.9)	1637 (18.5)	383 (20.4)	560 (20.0)	355 (18.3)	207 (15.0)	132 (15.3)
$75 K or more	13567 (44.5)	2120 (23.9)	598 (31.9)	691 (24.7)	419 (21.6)	264 (19.2)	148 (17.2)
Decline to answer	1881 (6.2)	412 (4.7)	82 (4.4)	107 (3.8)	110 (5.7)	60 (4.4)	53 (6.1)
Employed, n (%)	22073 (72.4)	5014 (56.6)	1208 (64.4)	1638 (58.5)	1058 (54.6)	706 (51.3)	404 (46.9)
Has health insurance, n (%)	27719 (90.9)	8030 (90.7)	1764 (94.0)	2557 (91.3)	1747 (90.1)	1219 (88.6)	743 (86.2)
Body mass index, n (%)							
Underweight (< 18.5)	732 (2.4)	256 (2.9)	48 (2.6)	68 (2.4)	59 (3.0)	48 (3.5)	33 (3.8)
Normal weight (18.5–24.9)	11476 (37.7)	2540 (28.7)	580 (30.9)	808 (28.8)	543 (28.0)	391 (28.4)	218 (25.3)
Overweight (25.0–29.9)	9716 (31.9)	2258 (25.5)	550 (29.3)	745 (26.6)	449 (23.2)	328 (23.8)	186 (21.6)
Obese (30.0–39.9)	6308 (20.7)	2542 (28.7)	494 (26.3)	811 (29.0)	586 (30.2)	381 (27.7)	270 (31.3)
Morbidly obese (≥ 40)	1187 (3.9)	973 (11.0)	147 (7.8)	276 (9.9)	232 (12.0)	196 (14.2)	122 (14.2)
Decline to answer	1059 (3.5)	284 (3.2)	57 (3.0)	93 (3.3)	69 (3.6)	32 (2.3)	33 (3.8)
Smoking behavior, n (%)							
Never smoked	21814 (71.6)	4844 (54.7)	1095 (58.4)	1518 (54.2)	1072 (55.3)	682 (49.6)	477 (55.3)
Former smoker	5546 (18.2)	2275 (25.7)	466 (24.8)	744 (26.6)	495 (25.5)	365 (26.5)	205 (23.8)
Current smoker	3118 (10.2)	1734 (19.6)	315 (16.8)	539 (19.2)	371 (19.1)	329 (23.9)	180 (20.9)
Alcohol use, n (%)	20441 (67.1)	5839 (66.0)	1268 (67.6)	1918 (68.5)	1258 (64.9)	879 (63.9)	516 (59.9)
Regularly exercised	22372 (73.4)	5192 (58.6)	1215 (64.8)	1707 (60.9)	1091 (56.3)	748 (54.4)	431 (50.0)
CCI, mean ± SD	0.14 ± 0.61	0.44 ± 1.08	0.36 ± 0.98	0.42 ± 1.08	0.48 ± 1.11	0.49 ± 1.16	0.51 ± 1.08
**Depression symptoms at the time of diagnosis**							
Depressed mood and other emotional problems, n (%)	-	7791 (88.0)	1593 (84.9)	2478 (88.5)	1718 (88.6)	1216 (88.4)	786 (91.2)
Eating pattern changes, n (%)	-	1859 (21.0)	319 (17.0)	570 (20.3)	462 (23.8)	318 (23.1)	190 (22.0)
Sleep pattern changes, n (%)	-	3876 (43.8)	730 (38.9)	1231 (43.9)	906 (46.7)	642 (46.7)	367 (42.6)
Mental changes, n (%)	-	4340 (49.0)	748 (39.9)	1362 (48.6)	992 (51.2)	723 (52.5)	515 (59.7)
Social problems, n (%)	-	2810 (31.7)	481 (25.6)	854 (30.5)	628 (32.4)	503 (36.6)	344 (39.9)
Physical problems, n (%)	-	1342 (15.2)	251 (13.4)	433 (15.5)	293 (15.1)	230 (16.7)	135 (15.7)
**Anxiety and sleep problems**							
Diagnosed with anxiety total^c^ n (%)	1668 (5.5)	6481 (73.2)	1157 (61.7)	2007 (71.7)	1468 (75.7)	1114 (81.0)	735 (85.3)
Diagnosed with any sleep problem, n (%)	1995 (6.5)	3531 (39.9)	471 (25.1)	1073 (38.3)	866 (44.7)	646 (46.9)	475 (55.1)
**Prescription use**							
For anxiety total^c^, n (%)	815 (2.7)	3892 (44.0)	708 (37.7)	1183 (42.2)	897 (46.3)	661 (48.0)	443 (51.4)
For sleep total^d^, n (%)	715 (2.3)	1536 (17.4)	200 (10.7)	488 (17.4)	363 (18.7)	276 (20.1)	209 (24.2)
**GAD-7 scores**							
GAD-7 score, mean ± SD	1.16 ± 2.10	8.00 ± 5.57	3.10 ± 3.26	6.33 ± 3.99	9.01 ± 4.41	12.16 ± 4.59	15.15 ± 4.84

^a^Respondents with depression diagnosis: those who self-reported physician diagnosis of depression and reported experiencing depression in the past 12 months

^b^Respondents without depression diagnosis: those who had no self-reported physician diagnosis of depression, reported not experiencing depression in the past 12 months, and had PHQ-9 scores ≤ 4

^c^Anxiety total: Anxiety, generalized anxiety, social anxiety, PTSD, OCD, panic disorder, phobias

^d^Sleep total: Sleep difficulties, insomnia, sleep apnea

^*^P < 0.001 for all variables compared between ‘with depression diagnosis (total)’ vs. ‘without depression diagnosis’ groups

^#^*P* < 0.001 for all variables across all severity groups using an overall omnibus test, except “physical problems” for which *P* = 0.176

*CCI* Charlson comorbidity index, *GAD-7* Generalized anxiety disorder-7 scale, *OCD* Obsessive–compulsive disorder, PHQ-9 The Patient Health Questionnaire 9, *PTSD* Post-traumatic stress disorder, SD Standard deviation

### Depression symptoms and anxiety and sleep problems

The most frequent depression symptoms reported by respondents with a depression diagnosis were depressed mood and other emotional problems (88.0%), followed by mental changes (e.g., forgetfulness, difficulty thinking, difficulty concentrating; 49.0%), and sleep pattern changes (43.8%; Table 1). A higher proportion of respondents with severe symptoms had depressed mood and other emotional problems (91.2% vs. 84.9%; *P* < 0.001), mental changes (59.7% vs. 39.9%; *P* < 0.001), and sleep pattern changes (42.6% vs. 38.9%; *P* < 0.001) compared to those with no/minimal symptoms.

A significantly higher proportion of respondents with a depression diagnosis (total) were diagnosed with anxiety (73.2% vs. 5.5%; *P* < 0.001) and used prescription medications for anxiety (44.0% vs. 2.7%; *P* < 0.001) than respondents without depression diagnosis (Table 1). A similar trend was observed in respondents with a depression diagnosis (total) with regard to being diagnosed with sleep problems (any; 39.9% vs. 6.5%; *P* < 0.001) and use of prescription medications for sleep problems (17.4% vs. 2.3%; *P* < 0.001) compared to respondents without depression diagnosis (Table 1). The proportion of respondents diagnosed with anxiety or any sleep problems and using prescription medications for anxiety or sleep increased with increasing severity of depression.

Mean GAD-7 scores were higher in respondents with a depression diagnosis (total) (8.0) as well as those with no/minimal symptoms (3.1) than respondents without depression diagnosis (1.16). GAD-7 scores increased (ie, worsened) with increasing severity of depression symptoms (Table 1).

Similarly, a higher proportion of respondents with depression diagnosis (total) reported more sleep problems than respondents without any depression diagnosis (all *P* < 0.001; Supplementary Table 2). A higher proportion of those with no/minimal symptoms reported difficulty falling asleep (35.6% vs. 16.1%) and poor sleep quality (23.2% vs. 10.8%) than respondents without any depression diagnosis. The proportion experiencing sleep problems increased with increasing severity of depression. (Supplementary Table 2).

### Bivariate analyses

SF-36v2 summary scores (MCS and PCS) and all eight health domains, SF-6D, EQ-5D utility score and EQ VAS scores were lower in those with depression diagnosis (total) than those without depression diagnosis (all *P* < 0.001). These scores were also found to be lower in respondents with no/minimal symptoms than those without depression diagnosis. The scores worsened with increased severity (all *P* < 0.001) (Supplementary Table 3). Absenteeism, presenteeism, overall work impairment, and activity impairment scores were significantly higher for those with depression diagnosis (total) compared with those without depression diagnosis (all *P* < 0.001). The WPAI scores further worsened with increasing disease severity (all *P* < 0.001) (Supplementary Table 4). The number of HCP visits, ER visits, and hospitalizations in the last six months were significantly higher for those with depression diagnosis (total) than those without depression diagnosis (all *P* < 0.001) (Supplementary Table 5). Additionally, HRU increased significantly with an increase in disease severity.

### Multivariable analyses

**Fig. 2:**
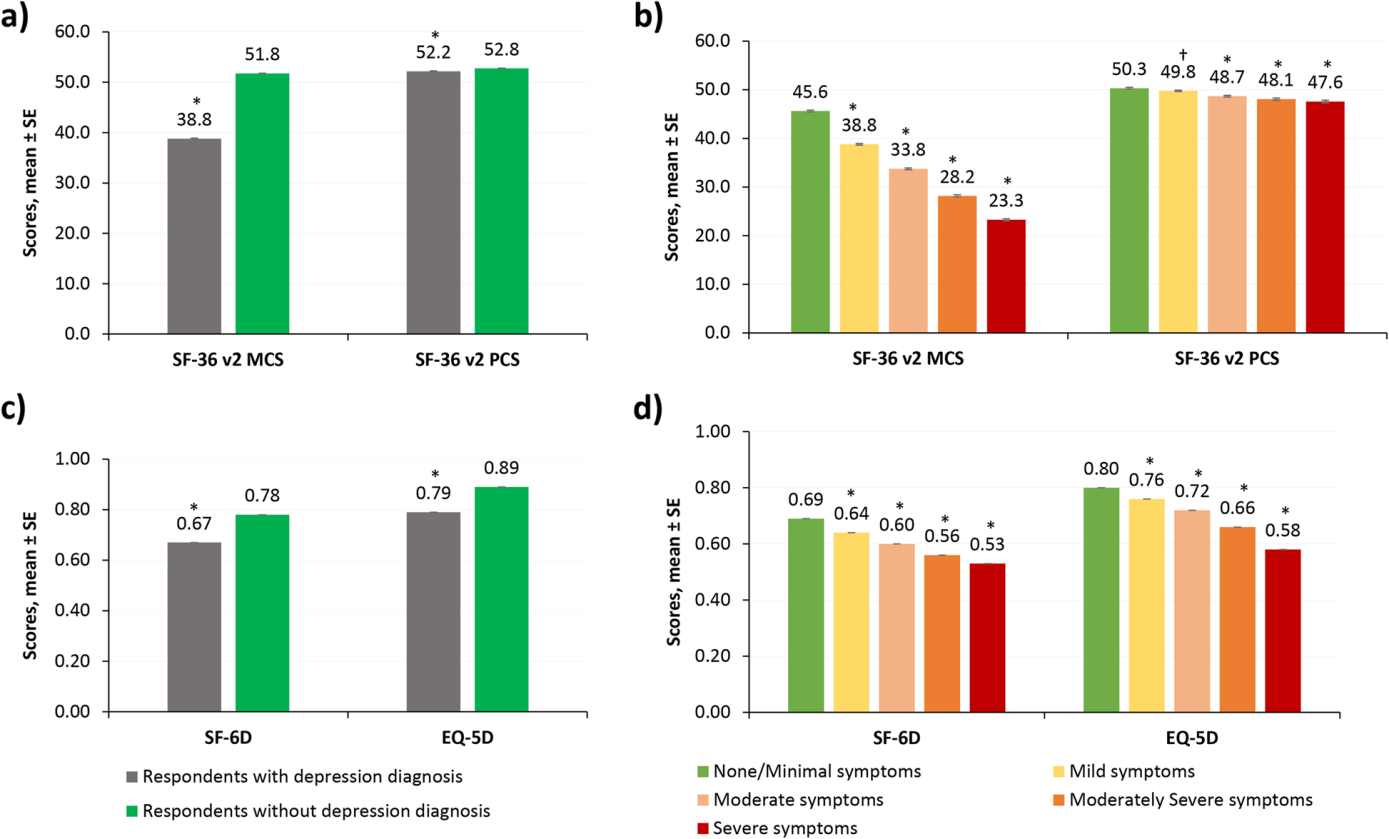
HRQoL outcomes among respondents with and without depression diagnosis and across severity groups – Multivariable results Results are based on generalized linear regression models controlling for demographics, health characteristics, and comorbidities as covariates. Respondents with depression diagnosis: those who self-reported physician diagnosis of depression and reported experiencing depression in the past 12 months. Respondents without depression diagnosis: those who had no self-reported physician diagnosis of depression, reported not experiencing depression in the past 12 months, and had PHQ-9 scores ≤ 4. In panels (b) and (d), the five groups are based on depression severity (PHQ-9 scores). *P-value < 0.001: comparison vs without depression diagnosis group in sub-figures a and c; comparison vs no/minimal symptoms severity group in sub-figures b and d. †*P*-value < 0.050 vs no/minimal symptoms severity group. Confidence bars represent standard error of the mean score. EQ-5D, EuroQol 5-Dimension; HRQoL, health-related quality of life; MCS, Mental Component Summary; PCS, Physical Component Summary; PHQ-9, the Patient Health Questionnaire 9; SF-36v2, the Medical Outcomes Study 36-Item Short Form Survey Instrument version 2; SF-6D, Short-Form 6 Dimensions

Survey respondents with depression diagnosis (total) had poorer MCS (38.8 vs. 51.8, *P* < 0.001) and PCS (52.2 vs. 52.8, *P* < 0.001) scores than survey respondents without depression diagnosis (Fig. 2a). MCS and PCS scores decreased with increasing severity of depression symptoms (Fig. 2b). Respondents with severe depression symptoms reported the greatest impairment on the MCS (23.3 vs. 45.6, *P* < 0.001) and PCS (47.6 vs. 50.3, *P* < 0.001) scores compared to those with no/minimal symptoms. Survey respondents with depression diagnosis (total) had worse health utilities (SF-6D: 0.67 vs. 0.78; EQ-5D: 0.79 vs. 0.89, both *P* < 0.001), and EQ VAS (70.5 vs. 81.0, *P* < 0.001) scores than survey respondents without depression diagnosis (Fig. 2c). Similarly, SF-6D utility score, EQ-5D utility score, and EQ VAS scores worsened with increasing severity (all *P* < 0.001; Fig. 2d).

**Fig. 3:**
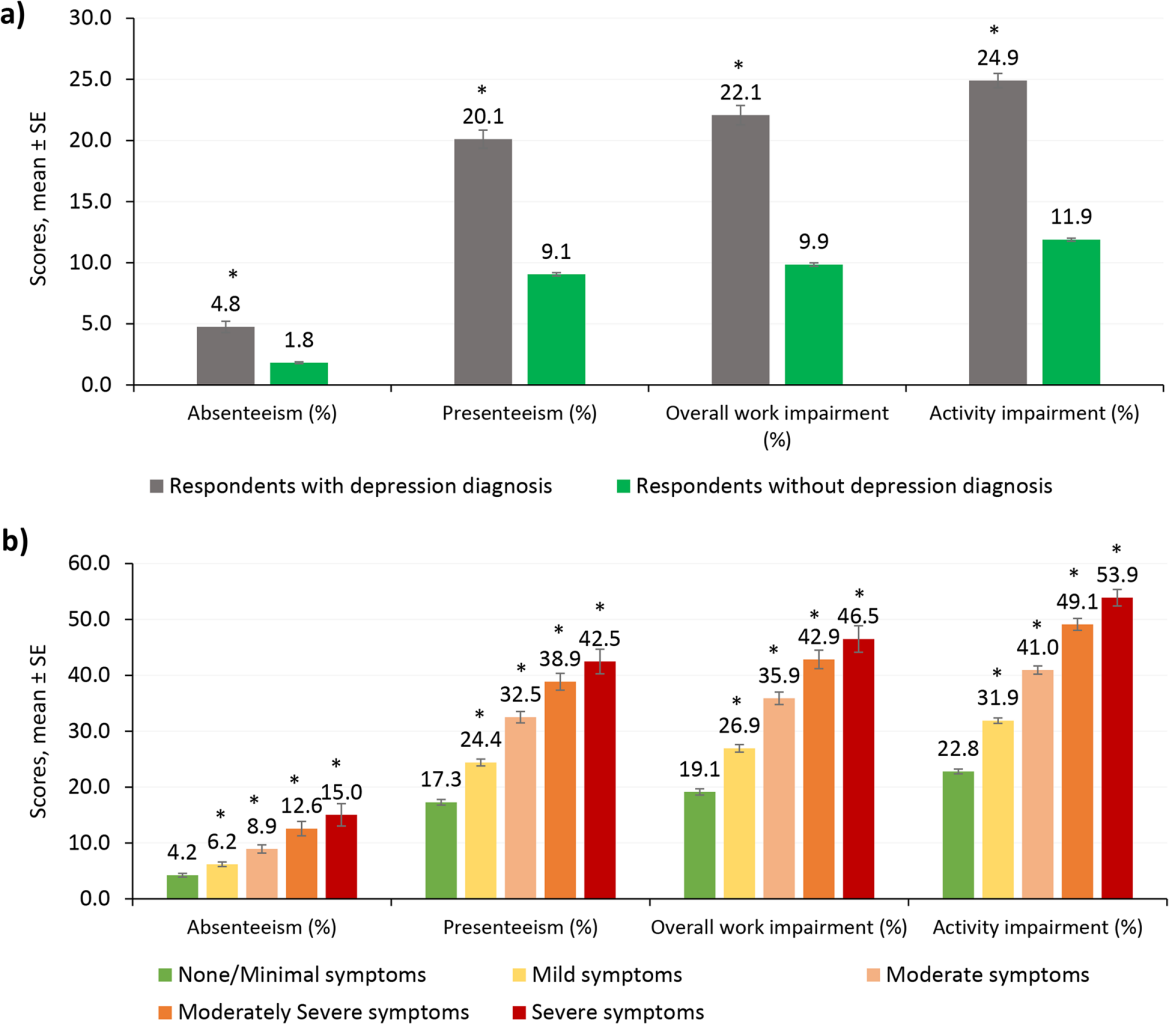
WPAI scores among respondents with and without depression diagnosis and across severity groups – Multivariable results Results are based on generalized linear regression models controlling for demographics, health characteristics, and comorbidities as covariates. Respondents with depression diagnosis: those who self-reported physician diagnosis of depression and reported experiencing depression in the past 12 months. Respondents without depression diagnosis: those who had no self-reported physician diagnosis of depression, reported not experiencing depression in the past 12 months, and had PHQ-9 scores ≤ 4. In panel (b), the five groups are based on depression severity (PHQ-9 scores). *P-value < 0.001: comparison vs without depression diagnosis group in sub-figure a; comparison vs no/minimal symptoms severity group in sub-figure b. Confidence bars represent standard error of the mean score. PHQ-9, the Patient Health Questionnaire 9; WPAI, work productivity and activity impairment

Respondents with depression diagnosis reported more absenteeism (4.8% vs. 1.8%), presenteeism (20.1% vs. 9.1%), and overall work impairment (22.1% vs. 9.9%) when employed than respondents without depression diagnosis (all *P* < 0.001) (Fig. 3a). Respondents with depression diagnosis reported greater activity impairment (24.9% vs. 11.9%, *P* < 0.001) than those without depression diagnosis. As severity increased, absenteeism, presenteeism, overall work impairment, and activity impairment increased (all *P* < 0.001) compared to those with no/minimal symptoms (Fig. 3b). Respondents with depression diagnosis reported greater number of HCP visits (3.6 vs. 2.4), ER visits (0.14 vs. 0.09), and hospitalizations (0.08 vs. 0.04) than respondents without depression diagnosis (all *P* < 0.001) in the last 6 months (Table 2). In general, the mean number of HCP visits, ER visits, and hospitalizations in the last six months increased with increasing severity. Respondents with severe depression symptoms reported significantly higher HRU compared to those with no/minimal symptoms (*P* < 0.050).

**Table 2:** HRU among respondents with depression versus without depression diagnosis and across severity groups – Multivariable results

**HRU in the past 6 months**	**Respondents without depression diagnosis**^**b**^ *n* = 30,478	**Respondents with depression diagnosis**^**a**^					
		**Total (all symptom levels)** *n* = 8853	**No/minimal symptoms** *n* = 1876	**Mild symptoms** *n* = 2801	**Moderate symptoms** *n* = 1938	**Moderately severe symptoms** *n* = 1376	**Severe symptoms** *n* = 862
Healthcare provider visits, mean ± SE	2.41 ± 0.03	3.61 ± 0.08^‡^	5.46 ± 0.17	5.60 ± 0.14	5.78 ± 0.17	6.23 ± 0.22**	6.50 ± 0.3**
ER visits, mean ± SE	0.09 ± 0	0.14 ± 0.01^‡^	0.25 ± 0.02	0.23 ± 0.01	0.26 ± 0.02	0.31 ± 0.02*	0.34 ± 0.03**
Hospitalizations, mean ± SE	0.04 ± 0	0.08 ± 0.01^‡^	0.11 ± 0.01	0.10 ± 0.01	0.11 ± 0.01	0.13 ± 0.02	0.16 ± 0.02*

Results are based on generalized linear regression models controlling for demographics, health characteristics, and comorbidities as covariates

^a^Respondents with depression diagnosis: those who self-reported physician diagnosis of depression and reported experiencing depression in the past 12 months

^b^Respondents without depression diagnosis: those who had no self-reported physician diagnosis of depression, reported not experiencing depression in the past 12 months, and had PHQ-9 scores ≤ 4

^*^P-value < 0.050 for comparison vs ‘no/minimal symptoms’ severity group

^**^P-value < 0.010 for comparison vs ‘no/minimal symptoms’ severity group

^‡^*P*-value < 0.001 for comparison vs ‘without depression diagnosis’ group

*ER* Emergency room, *HRU* Healthcare resource utilization, *SE* Standard error

## Discussion

The current study provides important insights into the humanistic and economic burden associated with diagnosed depression in the US. The study findings demonstrate that notable differences exist in terms of symptom burden, QoL, WPAI, and HRU between respondents with depression diagnosis (self-reported) versus those without depression diagnosis, as well as across depression groups based on severity.

The sociodemographic characteristics of respondents with depression diagnosis correlated with previous studies in terms of high prevalence in women and high rates of comorbidities, most notably anxiety, compared to those without depression diagnosis [26, 27]. In the current study, 73.2% of subjects with depression reported a diagnosis of anxiety, which is in line with previous research that reported significant anxiety in majority of patients with depression (85%) [27].

In our study, of those respondents with depression diagnosis (*n* = 8853), 47.2% had moderate-to-severe symptoms and 21.2% had no/minimal symptoms of depression, at the time of the survey. Respondents with no/minimal symptoms of depression, as determined by the PHQ-9 scores at the time of survey, indicate potential remission or that depression had resolved. However, these respondents still reported a higher burden of anxiety and sleep issues than respondents without a depression diagnosis. These findings are consistent with published studies which indicate that patients’ symptomatic recovery could be misleading as they may continue to experience reduced QoL and disabling residual symptoms, while in remission [9, 28–30].

Consistent with previously published studies, respondents with depression diagnosis had poorer HRQoL (with lower scores on MCS, PCS, SF-6D, and EQ-5D scores) compared to those without depression diagnosis [7, 31, 32]. In the STAR*D study approximately 50% of the patients with depression reported experiencing “severely impaired” QoL even after antidepressant treatment [9]. The minimal important difference (MID) of 3 points on the MCS [33] was exceeded in the present study (with depression diagnosis: 38.8; without depression diagnosis: 51.8; *P* < 0.001), further confirming poorer mental health in respondents with depression; however, although PCS was significantly different between respondents with depression diagnosis versus without depression diagnosis (52.2 vs 52.8; *P* < 0.001), the MID of 3 points on the PCS [33] was not reached. Respondents with higher depressive symptom severity also reported a higher burden of illness across various health indices as compared with the no/minimal symptom severity group. A similar trend was reported by a study in Europe that showed reduced HRQoL as assessed by SF-12 and the EQ-5D utility index scores in patients with MDD [5]. Taken together, the data are suggestive of the substantial humanistic burden and need for specific interventions to improve HRQoL in patients with depression.

The current study also demonstrated that respondents with depression diagnosis reported greater work productivity losses when employed than respondents without depression diagnosis. These results are in agreement with previously published data that demonstrated higher presenteeism, absenteeism, overall work impairment, and activity impairment in those with depression versus those without depression [31, 34, 35]. We also observed higher presenteeism than absenteeism in the current study, which is consistent with previous research [31], where presenteeism could be identified as the primary contributor to work productivity loss. Further, studies by Beck et al. reported that even minimal levels of depression symptoms were associated with loss of work productivity [13, 36]. Our findings are in line with prior research that demonstrated increase in WPAI with the severity of the disease [12, 34, 36].

In the current study, respondents with depression diagnosis had a greater number of HCP visits, ER visits, and hospitalizations than those without depression diagnosis. These findings are in accord with prior research that reported higher HRU in those with depression compared to controls [31, 32]. In our study, the frequency of hospitalizations and ER visits increased with increase in the severity of depression, similar to previous research [12]. Collectively these observations suggest that respondents with a depression diagnosis may incur higher direct and indirect costs than those without depression.

Recently, research has shown the impact of the pandemic on mental health, and the growing prevalence of depression [37, 38]. Thus, the treatment of depression has become an even more critical issue, from both humanistic and economic perspectives.

### Study implications

Our study findings confirm that individuals with depression diagnosis have substantial humanistic and economic burden and provide unique insights into the varying burden experienced among individuals with different severity of disease. This increases our understanding about the overall effect of depression on the HRQoL of patients as well as the burden of lost work productivity on employers and increased resource utilization on payers. Substantially higher burden of illness even among respondents with minimal symptoms (vs. those without depression) is suggestive that they might not be exhibiting symptoms on common and validated scales (such as PHQ-9 in this case). It is important to note that the evaluation of treatment efficacy typically focuses on depression symptom scales that are an important part of treatment, but do not fully capture patient-centric concepts related to well-being. Considering the impact that depression has on various aspects of patients’ lives, there is an additional need to treat patients focusing on core symptom resolution as well as overall and broader well-being, including functional improvement and minimizing residual symptoms [29, 39–42]. Additionally, it is important to broaden the routine outcome monitoring systems [43] to effectively capture patients’ experiences, perspectives, needs, and priorities and incorporating them into the treatment approach for better outcomes [42]. The combination of psychotherapy and pharmacotherapy has already shown to improve QoL and work productivity in patients diagnosed with depression [35, 44, 45]. Observations from this study provide insights for future research, while reinforcing the widespread need for novel, effective, and accessible treatment options for depression considering the negative impact of increasing depression severity on outcomes.

### Strengths and limitations

The sampling methodology utilized in the NHWS is designed to generate a representative sample of the US’ general population. However, results from this study may not be generalizable to the US’ population of patients diagnosed with depression. As an internet-based survey, similar to other patient-reported surveys, this approach likely underrepresents people with no access to or lack of comfort with online administration, less healthy elderly people, institutionalized patients, and those with low health literacy, severe comorbidities, and disabilities. The self-reported nature of the NHWS is also associated with potential corresponding biases such as recall and self-presentation biases. To reduce recall bias, shorter and recent timeframes for questions were chosen (e.g., HRU in the past 6 months). Moreover, the survey content was thoroughly reviewed with the study team to avoid any self-presentation bias. Physician records were not reviewed as part of the current study and therefore physician-diagnosed depression could not be verified. Thus, patients’ self-reported diagnosis of depression was recorded, which may have created a bias in the results obtained. Further, associations between depression and outcomes (e.g., HRU, HRQoL) may be complex due to the presence of comorbidities, which might have affected the results; these effects were controlled by including potential comorbidities as covariates in the multivariable analyses. However, there may be other confounders not collected in the current study.

PHQ-9, a well-validated and commonly used screening and monitoring scale, was used as one of the eligibility criteria and to classify the severity of depression. Previously published studies stated that PHQ-9 can be considered for screening but PHQ-9 scores above cut-off should be interpreted with caution or should be coupled with other assessment tools for definitive diagnosis in patients with major depression [46, 47]. Patients may rate high on the scale total score without having elevated core symptoms of depression. Additionally, demographic, cultural, and religious backgrounds may impact how a patient answers certain questions. No strong causal conclusions can be drawn between MDD and outcomes due to the cross-sectional nature of this study. Hence, a longitudinal study would be needed to reduce bidirectional effects.

## Conclusions

The overall study results demonstrate that respondents with depression diagnosis (self-reported) experienced lower HRQoL, higher WPAI, and greater HRU compared to those without depression diagnosis. The humanistic and economic burden of depression increased with severity of illness. Furthermore, the study results highlight that the burden of depression remains high, even among those experiencing minimal severity/symptoms of depression. Although such patients may show symptomatic relief/remission of symptoms, they continue to experience lower QoL compared to controls. These results indicate the need for novel and effective treatments that help improve patients’ wellness and functioning for this psychiatric disorder. Further, while treating patients, it is important to focus not only on the resolution of depressive symptoms but also on the overall well-being, including functional improvement.

## Supplementary Information

**Additional file 1: Supplementary Table 1.** List of variables/outcomes collected. **Supplementary Table 2.** Sleep problems experienced by respondents with depression versus without depression diagnosis and across severity groups. **Supplementary Table 3.** HRQoL outcomes among respondents with and without depression diagnosis and across severity groups – Bivariate results. **Supplementary Table 4.** WPAI scores among respondents with and without depression diagnosis and across severity groups – Bivariate results. **Supplementary Table 5.** HRU among respondents with depression versus without depression diagnosis and across severity groups – Bivariate results.

## Data Availability

The data that support the findings of this study are available from Cerner Enviza but restrictions apply to the availability of these data, which were used under license for the current study, and so are not publicly available. Data can be made available for non-commercial use from the authors upon reasonable request and with permission of Cerner Enviza.

## Abbreviations

ANOVA: Analysis of Variance
BMI: Body mass index
CCI: Charlson Comorbidity Index
EQ-5D-5L: EuroQol 5-Dimension Health Questionnaire
EQ VAS: EuroQol Visual Analogue Scale
ER: Emergency room
GAD-7: Generalized Anxiety Disorder-7 scale
GLMs: Generalized linear models
HCP: Healthcare provider
HRQoL: Health related quality of life; HRU: Healthcare resource utilization
MCS: Mental component summary
MDD: Major depressive disorder
MID: Minimal important difference
NHWS: National Health and Wellness Survey
PCS: Physical component summary
PHQ-9: Patient Health Questionnaire-9
QoL: Quality of life
SF-36v2: Short Form Survey Instrument version 2
SF-6D: Short-Form-6 Dimensions
STAR*D: Sequenced Treatment Alternatives to Relieve Depression
US: United States
WPAI: Work productivity and activity impairment
