# Supplementary material for: Humanistic and economic burden associated with depression in the United States: a cross-sectional survey analysis
Source: BMC Psychiatry. 2022 Aug 11;22:542. doi: 10.1186/s12888-022-04165-x (PMC9367058; doi:10.1186/s12888-022-04165-x)
Supplement: Supplementary file 1 — Additional file 1: Supplementary Table 1. List of variables/outcomes collected. Supplementary Table 2. Sleep problems experienced by respondents with depression versus without depression diagnosis and across severity groups. Supplementary Table 3. HRQoL outcomes among respondents with and without depression diagnosis and across severity groups – Bivariate results. Supplementary Table 4. WPAI scores among respondents with and without depression diagnosis and across severity groups – Bivariate results. Supplementary Table 5. HRU among respondents with depression versus without depression diagnosis and across severity groups – Bivariate results. [file 12888_2022_4165_MOESM1_ESM.docx]

**Supplementary Table 1 List of variables/outcomes collected**

| **Variables measured** | **Data collected/scale or questionnaire utilized** |
| --- | --- |
| Demographics and health characteristics | **Age**, **gender** (male or female), **employment status** (employed [full-time or part-time] or unemployed), **race/ethnicity** (US only; non-Hispanic white, Hispanic, non-Hispanic black, other), **marital status** (married/living with partner or not-married/living with partner [divorced, separated, never married or widowed]), **education** (university degree or less than university degree), **household income** (<$25K, $25k to <50K, $50k to <75K, $75K or more, or decline to answer) **insurance status type** (e.g., insured or uninsured), **BMI** (underweight, normal, overweight, obese, morbidly obese or decline to answer), **smoking status** (currently smoke, former smoker vs. never smoker), **alcohol use** (daily, weekly, monthly or abstain), and **exercise behavior** (number of days in the past month exercised vigorously for 20 mins)  Charlson Comorbidity Index, diagnosed comorbidities (e.g., diabetes, hypertension, asthma, anxiety, migraine) |
| Depression severity | The PHQ-9[25] measures frequency of depression symptoms over the last 2 weeks, with items scored on a 4-point scale (not at all=0 to nearly every day=3). Items are summed to a total score of 0-27. Scores of 5, 10, 15, and 20 represent cutoff points for mild, moderate, moderately severe, and severe symptoms. Scores 0-4 indicate no/minimal symptoms. |
| Anxiey | The GAD-7 anxiety scale[19] is a 7-item general anxiety measure. Individuals rate how bothered they have been by different anxiety symptoms over the last 2 weeks, with items scored from 0 to 3, providing a 0-21 severity score for anxiety. Scores of 5, 10, and 15 represent cutoff points for mild, moderate, and severe anxiety, respectively. Diagnosis of anxiety and prescription use for anxiety were self-reported. |
| Depression symptoms | Depression symptoms included: self-reported depressed mood and other emotional problems (e.g., hopelessness, tiredness, anxiety), eating pattern changes (e.g., appetite loss, weight loss, overeating, weight gain), sleep pattern changes (e.g., difficulty sleeping, oversleeping, waking up early), mental changes (e.g., forgetfulness, difficulty thinking, difficulty concentrating), social problems (e.g., isolation, alcohol problems, drug problems, sex problems), and physical problems (e.g., headaches, body aches, constipation). |
| Sleep problems | - Sleep problems included: self-reported difficulty falling asleep, difficulty staying awake, daytime sleepiness, leg cramps/leg problems, night sweats/hot flashes, poor quality of sleep, sleep apnea, waking up to go to the bathroom, waking up too early (such as before the alarm clock), waking during the night and not being able to get back to sleep, and waking up several times during the night. - Diagnosis of sleep problems, prescription use for sleep problems, as well as individual symptoms regularly experienced were self-reported. |
| HRQoL: SF-36v2 | SF-36v2[20] is a multipurpose, generic health status instrument comprising 36 questions. The instrument is designed to report on eight health domains (physical functioning, physical role limitations, bodily pain, general health, vitality, social functioning, emotional role limitations, and mental health). Two summary scores are calculated: PCS and MCS, that are normed to a mean of 50 and a standard deviation of 10. Higher scores indicate better health status. |
| HRQoL: SF-6D | The SF-6D[21] is a preference-based single index measure for health using general population values. The SF-6D index has interval scoring properties and yields summary scores on a theoretical 0–1 scale. Higher scores indicate better health status. |
| HRQoL: EQ-5D-5L and EQ VAS | - The EQ-5D-5L[21] measures quality of life in five dimensions (mobility, self-care, usual activities, pain/discomfort, and anxiety/depression). Lower overall scores on the EQ-5D-5L are indicative of higher disability. - The EQ VAS asks respondents to indicate on a line their self-rated health, with the endpoints of the line being 'Best imaginable health state' and 'Worst imaginable health state'. |
| WPAI | WPAI[24] questionnaire, a 6-item validated instrument, consists of four metrics: absenteeism (the percentage of work time missed because of one's health in the past seven days), presenteeism (the percentage of impairment experienced while at work in the past seven days because of one's health), overall work impairment (an overall impairment estimated: combination of absenteeism and presenteeism), and activity impairment (the percentage of impairment in daily activities because of one's health in the past seven days). Only respondents who report being employed provided data for absenteeism, presenteeism, and overall work impairment. All respondents provided data for activity impairment. |
| Healthcare resource use | Healthcare utilization was defined by the number of traditional healthcare provider visits, the number of ER visits, and the number of times hospitalized in the past six months. |

EQ-5D-5L, EuroQol 5-Dimension Health Questionnaire; EQ-VAS, EuroQol Visual Analogue Scale; ER, emergency room; GAD-7, the Generalized Anxiety Disorder-7 scale; HRQoL, health-related quality of life; MCS, Mental Component Summary; PCS, Physical Component Summary; PHQ-9, the Patient Health Questionnaire 9; SF-36v2, the Medical Outcomes Study 36-Item Short Form Survey Instrument version 2; SF-6D, Short-Form 6 Dimensions; WPAI, Work Productivity and Activity Impairment.

**Supplementary Table 2 Sleep problems experienced by respondents with depression versus without depression diagnosis and across severity groups**

| **Variable, n (%)** | **Respondents without depression diagnosis^b^**  n=30478 | **Respondents with depression diagnosis^a^** | | | | | | |
| --- | --- | --- | --- | --- | --- | --- | --- | --- |
|  |  | **Total (all symptom levels)**  n=8853 | **No/minimal symptoms**  n=1876 | **Mild symptoms**  n=2801 | **Moderate symptoms**  n=1938 | **Moderately severe symptoms**  n=1376 | **Severe symptoms**  n=862 | |
| Difficulty falling asleep | 4912 (16.1) | 5058 (57.1) | 667 (35.6) | 1545 (55.2) | 1265 (65.3) | 926 (67.3) | | 655 (76.0) |
| Waking during the night and not being able to get back to sleep | 3655 (12.0) | 3493 (39.5) | 429 (22.9) | 1041 (37.2) | 866 (44.7) | 682 (49.6) | | 475 (55.1) |
| Waking up several times during the night | 4063 (13.3) | 3864 (43.6) | 476 (25.4) | 1170 (41.8) | 943 (48.7) | 751 (54.6) | | 524 (60.8) |
| Waking up too early | 4433 (14.5) | 3284 (37.1) | 419 (22.3) | 1009 (36.0) | 822 (42.4) | 603 (43.8) | | 431 (50.0) |
| Sleep apnea (temporary absence of breathing) | 1050 (3.4) | 1082 (12.2) | 164 (8.7) | 342 (12.2) | 235 (12.1) | 200 (14.5) | | 141 (16.4) |
| Leg cramps/leg problems | 1632 (5.4) | 1703 (19.2) | 186 (9.9) | 487 (17.4) | 423 (21.8) | 341 (24.8) | | 266 (30.9) |
| Waking up to go to bathroom | 5413 (17.8) | 3478 (39.3) | 519 (27.7) | 1125 (40.2) | 801 (41.3) | 613 (44.5) | | 420 (48.7) |
| Night sweats/hot flashes | 1936 (6.4) | 2111 (23.8) | 247 (13.2) | 641 (22.9) | 529 (27.3) | 388 (28.2) | | 306 (35.5) |
| Pain | 1385 (4.5) | 1920 (21.7) | 166 (8.8) | 532 (19.0) | 519 (26.8) | 395 (28.7) | | 308 (35.7) |
| Poor quality of sleep | 3300 (10.8) | 4200 (47.4) | 435 (23.2) | 1240 (44.3) | 1062 (54.8) | 848 (61.6) | | 615 (71.3) |
| Daytime sleepiness | 3266 (10.7) | 4395 (49.6) | 470 (25.1) | 1308 (46.7) | 1117 (57.6) | 882 (64.1) | | 618 (71.7) |
| Difficulty staying awake | 632 (2.1) | 1814 (20.5) | 117 (6.2) | 411 (14.7) | 482 (24.9) | 447 (32.5) | | 357 (41.4) |

^a^Respondents with depression diagnosis: those who self-reported physician diagnosis of depression and reported experiencing depression in the past 12 months).

^b^Respondents without depression diagnosis: those who had no self-reported physician diagnosis of depression, reported not experiencing depression in the past 12 months, and had PHQ-9 scores ≤4.

PHQ-9, the Patient Health Questionnaire 9.

**Supplementary Table 3 HRQoL outcomes among respondents with and without depression diagnosis and across severity groups – Bivariate results**

| **Outcomes,** mean ± SD | **Respondents without depression diagnosis^b*^**  n=30478 | **Respondents with depression diagnosis^a^** | | | | | |
| --- | --- | --- | --- | --- | --- | --- | --- |
|  |  | **Total (all symptom levels)***  n=8853 | **No/minimal symptoms^#^**  n=1876 | **Mild symptoms^#^**  n=2801 | **Moderate symptoms^#^**  n=1938 | **Moderately severe symptoms^#^**  n=1376 | **Severe symptoms^#^**  n=862 |
| **SF-36v2** | | | | | | | |
| MCS | 52.68 ± 7.10 | 35.98 ± 11.49 | 46.46 ± 8.17 | 39.06 ± 8.69 | 33.60 ± 8.70 | 27.49 ± 8.80 | 22.06 ± 9.02 |
| PCS | 53.63 ± 7.07 | 49.17 ± 10.80 | 51.51 ± 8.92 | 49.80 ± 10.47 | 48.40 ± 11.20 | 47.48 ± 11.55 | 46.42 ± 12.12 |
| Bodily pain | 53.40 ± 8.09 | 45.42 ± 10.12 | 49.58 ± 8.99 | 46.45 ± 9.49 | 44.24 ± 9.78 | 42.41 ± 10.17 | 40.49 ± 11.00 |
| General health | 53.71 ± 7.34 | 48.15 ± 10.46 | 51.41 ± 8.79 | 49.18 ± 9.79 | 47.42 ± 10.43 | 45.44 ± 11.20 | 43.65 ± 11.85 |
| Vitality | 53.05 ± 7.45 | 45.64 ± 11.17 | 50.50 ± 8.93 | 47.32 ± 10.29 | 44.39 ± 10.91 | 41.60 ± 11.75 | 38.80 ± 11.86 |
| Social functioning | 54.11 ± 7.93 | 40.41 ± 9.70 | 48.81 ± 8.09 | 42.32 ± 7.99 | 38.13 ± 7.88 | 34.31 ± 7.71 | 30.76 ± 7.24 |
| Mental health | 53.84 ± 8.28 | 43.50 ± 10.57 | 49.18 ± 9.54 | 44.96 ± 9.92 | 41.87 ± 9.81 | 39.21 ± 9.75 | 36.88 ± 10.28 |
| Role Emotional | 52.44 ± 7.84 | 39.97 ± 12.56 | 48.26 ± 9.58 | 42.61 ± 10.80 | 38.10 ± 11.24 | 32.99 ± 11.90 | 28.71 ± 12.13 |
| Role Physical | 52.94 ± 7.31 | 40.06 ± 10.88 | 48.19 ± 8.74 | 42.66 ± 9.02 | 38.23 ± 9.21 | 33.40 ± 9.22 | 28.73 ± 9.31 |
| Physical functioning | 53.06 ± 7.64 | 37.14 ± 10.47 | 46.53 ± 8.12 | 39.93 ± 8.00 | 35.09 ± 8.01 | 29.50 ± 7.59 | 24.42 ± 7.54 |
| **SF-6D Health state utility score** | 0.798 ± 0.112 | 0.618 ± 0.108 | 0.705 ± 0.107 | 0.639 ± 0.092 | 0.595 ± 0.082 | 0.553 ± 0.080 | 0.518 ± 0.085 |
| **ED-5D Health utility score** | 0.904 ± 0.109 | 0.726 ± 0.159 | 0.814 ± 0.120 | 0.763 ± 0.125 | 0.718 ± 0.143 | 0.648 ± 0.163 | 0.558 ± 0.175 |
| **EQ VAS score** | 82.81 ± 16.79 | 63.95 ± 22.40 | 74.71 ± 19.95 | 67.29 ± 20.50 | 61.60 ± 20.97 | 55.24 ± 21.94 | 48.85 ± 22.78 |

^a^Respondents with depression diagnosis: those who self-reported physician diagnosis of depression and reported experiencing depression in the past 12 months).

^b^Respondents without depression diagnosis: those who had no self-reported physician diagnosis of depression, reported not experiencing depression in the past 12 months, and had PHQ-9 scores ≤4.

*****For all outcomes, P<0.001 for comparisons of ‘with depression diagnosis (total)’ vs. ‘without depression diagnosis’ groups.

#For all outcomes, P<0.001 for comparisons of severity groups using an overall omnibus test.

EQ-5D, EuroQol 5-Dimension Health Questionnaire; EQ-VAS, EuroQol Visual Analogue Scale; HRQoL, health-related quality of life; MCS, mental component summary; PCS, physical component summary; PHQ-9, the Patient Health Questionnaire 9; SD, standard deviation; SF-36v2, the Medical Outcomes Study 36-Item Short Form Survey Instrument version 2; SF-6D, Short-Form 6 Dimensions.

**Supplementary Table 4 WPAI scores among respondents with and without depression diagnosis and across severity groups – Bivariate results**

| **Outcomes** | **Respondents without depression diagnosis^b*^**  n=30478 | **Respondents with depression diagnosis^a^** | | | | | |
| --- | --- | --- | --- | --- | --- | --- | --- |
|  |  | **Total (all symptom levels)***  n=8853 | **No/minimal symptoms^#^**  n=1876 | **Mild symptoms^#^**  n=2801 | **Moderate symptoms^#^**  n=1938 | **Moderately severe symptoms^#^**  n=1376 | **Severe symptoms^#^**  n=862 |
| **Absenteeism** | | | | | | | |
| Valid N | 21027 | 4802 | 1163 | 1571 | 1000 | 676 | 392 |
| % Mean ± SD | 1.96 ± 9.71 | 9.01 ± 20.03 | 4.49 ± 13.79 | 6.56 ± 16.66 | 9.78 ± 19.68 | 15.57 ± 26.29 | 18.94 ± 28.37 |
| **Presenteeism** | | | | | | | |
| Valid N | 20999 | 4721 | 1156 | 1552 | 987 | 654 | 372 |
| % Mean ± SD | 9.03 ± 18.81 | 28.99 ± 26.44 | 17.53 ± 22.97 | 25.01 ± 23.78 | 33.77 ± 25.25 | 41.35 ± 26.85 | 46.75 ± 28.29 |
| **Overall work impairment** | | | | | | | |
| Valid N | 20943 | 4708 | 1155 | 1549 | 983 | 650 | 371 |
| % Mean ± SD | 9.86 ± 20.04 | 32.00 ± 28.75 | 19.44 ± 24.93 | 27.62 ± 25.91 | 37.32 ± 27.51 | 45.70 ± 29.20 | 51.33 ± 30.12 |
| **Activity impairment** | | | | | | | |
| Valid N | 30478 | 8853 | 1876 | 2801 | 1938 | 1376 | 862 |
| % Mean ± SD | 11.87 ± 21.19 | 39.03 ± 29.03 | 23.04 ± 25.53 | 33.95 ± 26.91 | 43.27 ± 26.60 | 52.32 ± 26.92 | 59.55 ± 27.50 |

^a^Respondents with depression diagnosis: those who self-reported physician diagnosis of depression and reported experiencing depression in the past 12 months).

^b^Respondents without depression diagnosis: those who had no self-reported physician diagnosis of depression, reported not experiencing depression in the past 12 months, and had PHQ-9 scores ≤4.

*For all outcomes, P<0.001 for comparisons of ‘with depression diagnosis (total)’ vs. ‘without depression diagnosis’ groups.

#For all outcomes, P<0.001 for comparisons of severity groups using an overall omnibus test.

PHQ-9, the Patient Health Questionnaire 9; SD, standard deviation; WPAI, work productivity and activity impairment.

**Supplementary Table 5 HRU among respondents with depression versus without depression diagnosis and across severity groups – Bivariate results**

| **Outcomes** | **Respondents without depression diagnosis^b*^**  n=30478 | **Respondents with depression diagnosis^a^** | | | | | |
| --- | --- | --- | --- | --- | --- | --- | --- |
|  |  | **Total (all symptom levels)***  n=8853 | **No/minimal symptoms^#^**  n=1876 | **Mild symptoms^#^**  n=2801 | **Moderate symptoms^#^**  n=1938 | **Moderately severe symptoms^#^**  n=1376 | **Severe symptoms^#^**  n=862 |
| Healthcare provider visits in past 6 months, mean ± SD | 2.36 ± 3.46 | 6.40 ± 9.22 | 5.20 ± 7.18 | 6.00 ±9.62 | 6.43 ± 7.99 | 7.50 ± 10.30 | 8.44 ±11.72 |
| ER visits in the past 6 months, mean ± SD | 0.10 ± 0.82 | 0.34 ± 1.16 | 0.25 ± 1.03 | 0.28 ± 0.77 | 0.34 ± 1.02 | 0.44 ± 1.07 | 0.56 ± 2.33 |
| Hospitalizations in the past 6 months, mean ± SD | 0.05 ± 0.86 | 0.15 ± 0.85 | 0.13 ± 1.35 | 0.12 ± 0.58 | 0.14 ± 0.60 | 0.18 ± 0.60 | 0.23 ± 0.96 |

^a^Respondents with depression diagnosis: those who self-reported physician diagnosis of depression and reported experiencing depression in the past 12 months).

^b^Respondents without depression diagnosis: those who had no self-reported physician diagnosis of depression, reported not experiencing depression in the past 12 months, and had PHQ-9 scores ≤4.

*For all outcomes, P<0.001 for comparisons of ‘with depression diagnosis (total)’ vs. ‘without depression diagnosis’ groups.

#For all outcomes, P<0.001 for comparisons of severity groups, except “Hospitalizations in the past 6 months” for which P=0.009, using an overall omnibus test.

ER, emergency room; HRU, healthcare resource utilization; PHQ-9, the Patient Health Questionnaire 9; SD, standard deviation.
